# Gut Microbiota Modulation in CNS Disorders: Therapeutic Strategies and Implications for Drug Efficacy

**DOI:** 10.1007/s12035-026-06192-1

**Published:** 2026-10-15

**Authors:** Lamiaa A. Ahmed, Khaled F. Al-Massri

**Affiliations:** 1 Department of Pharmacology and Toxicology, Faculty of Pharmacy, Cairo University, Cairo, Egypt; 2 Department of Pharmacy and Biotechnology, Faculty of Medicine and Health Sciences, University of Palestine, Gaza, Palestine

**Keywords:** Dysbiosis, Microbiota-gut-brain axis, Prebiotics, Probiotics

## Abstract

Various central nervous system (CNS) disorders, including neurodegenerative diseases, depression, schizophrenia, epilepsy, and anxiety, present significant obstacles to public health because of their intricate causes and the scarcity of effective treatments. Recent studies have shown that gut microbiota (GM) and CNS communicate bidirectionally. Modulation of immune system, neurotransmitters, and neuroactive metabolites are only a few examples of how GM can significantly influence CNS. Dysbiosis or changes in composition and function of gut microbes play a contributing role in various CNS disorders. In these disorders, probiotics, prebiotics, fecal microbiota transplantation, antibiotics use, and interventions based on microbial metabolites have demonstrated beneficial effects in animal models and some clinical studies. By restoring microbial equilibrium, increasing microbial diversity, and promoting the generation of beneficial metabolites, these approaches can improve the efficacy of traditional medications while minimizing their side effects and the disease itself, based on preclinical evidence and emerging clinical studies. Nevertheless, several obstacles should be overcome before the clinical translation of these approaches, such as using of standardized methods for modulating GM, identifying specific microbial signatures linked to various CNS illnesses, and understanding the exact mechanisms of gut-brain communication. This review provides a novel integrated perspective by comprehensively clarifying the bidirectional interactions between CNS pharmacological agents and microbiota-based therapeutic strategies in different neurological and psychiatric disorders, offering a unified framework to improve traditional pharmacological therapies. Importantly, future studies should concentrate on exploring the mechanisms and the exact interconnections between GM and CNS problems to elaborate targeted therapies for these disorders.

**Graphical Abstract:**

**Figure:**
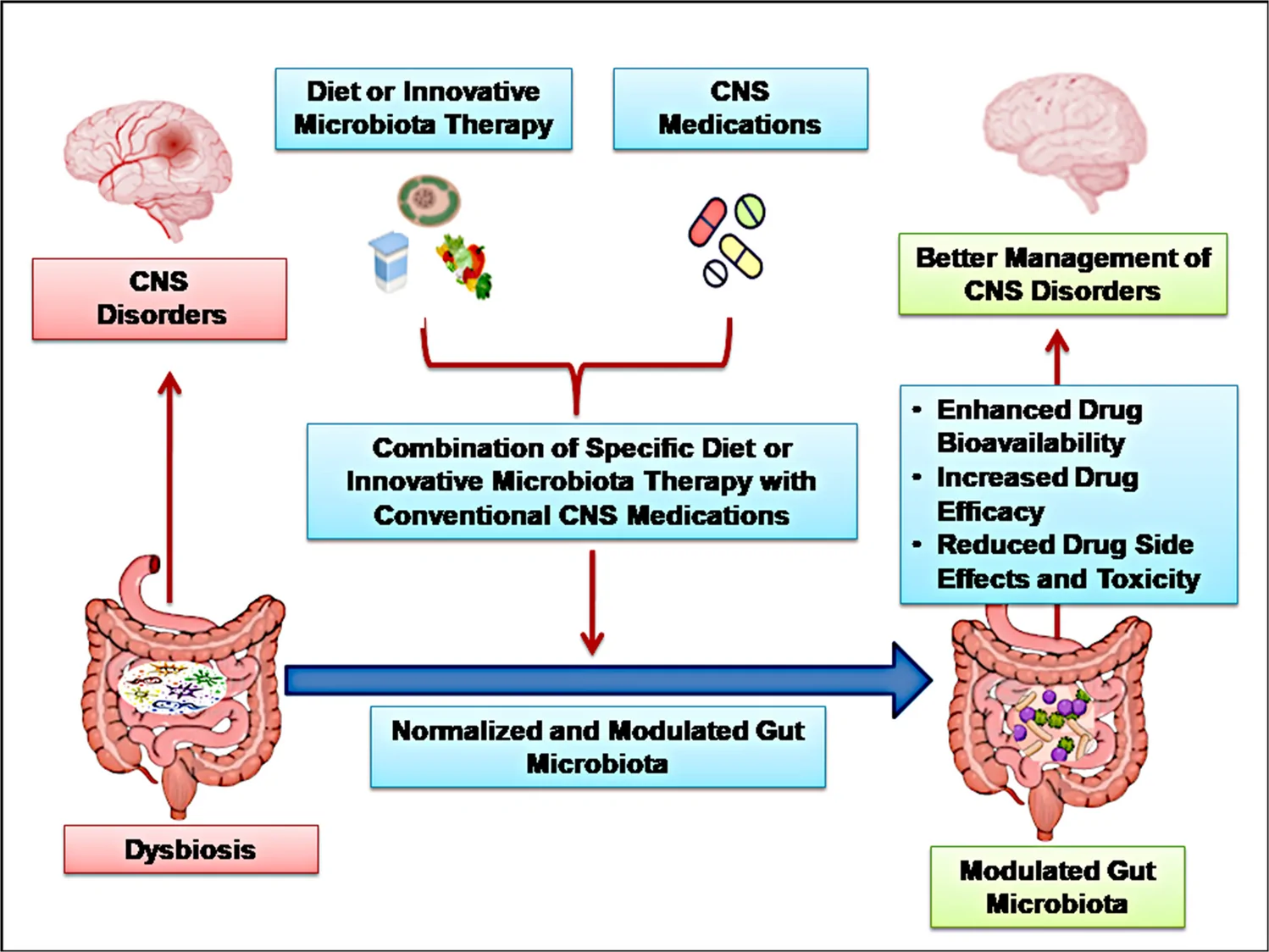

## Introduction

Various central nervous system (CNS) diseases present a significant risk to human health, where the mechanisms and management strategies of these diseases need to be further investigated [1]. Gut microbiota (GM) changes or dysbiosis are correlated with alterations of the structure of microbiota, aberrant microbial metabolites, breakdown of intestinal barrier, and increased risk of systemic inflammation, where all of these factors contribute to progression of CNS illnesses [2].

Importantly, the human body is housing a vast community of microorganisms, including approximately 10^13^–10^14^ microbes, that play a potential role in controlling and affecting several host physiology and disorders [3]. The majority of these microorganisms, or GM, live in the gastrointestinal system, where their composition is influenced by a number of circumstances. *Bacteroidetes*, *Firmicutes*, *Proteobacteria*, and *Actinobacteria* are the four main phyla that make up GM, whereas *Verrucomicrobia* and *Fusobacteria* are the two minor groups [4]. In order to keep the gut homeostasis and boost the host immune system, these commensal bacteria communicate with one another and the host gut epithelium. Notably, studies have shown that gut dysbiosis contributes to the development of several CNS disorders such as Alzheimer’s disease (AD), Parkinson’s disease (PD), amyotrophic lateral sclerosis (ALS), schizophrenia, depression, epilepsy, and anxiety [5–7]. Due to the bidirectional nature of gut-brain axis, the neurological disorders can also influence GM composition through impairment of the autonomic nervous system or inducing a status of chronic inflammation, which may affect gut environment and microbiota composition. However, this interpretation is hindered by limited studies which may require further research [7].

Nevertheless, despite increasing evidence on the influence of microbiota–gut–brain axis, the identification of effective targeted therapies remains a challenge due to the incomplete understanding of the detailed molecular mechanisms underlying microbiota-mediated effects in these disorders [8]. An increasing body of research points to a bidirectional way of communication between the digestive tract and the CNS, which is critical for the proper functioning of both systems. Irritable bowel syndrome is thought to be caused by mental health issues, such as anxiety, depression, schizophrenia, epilepsy, and neurodegenerative diseases and vice versa [9, 10].

Notably, changes in GM composition can impact the effectiveness of traditional medications used to treat a wide range of CNS disorders, including modulation of drug metabolism, systemic inflammatory status, and host–microbe interactions; however, the magnitude of these outcomes may differ according to the drug class and the stage of disease [11, 12]. Additionally, weight gain and other adverse effects of these medications may be caused by gut dysbiosis and alterations in the generated metabolites [13]. All of these aspects confirm the role of GM in worsening or alleviation of CNS conditions. GM modulation may also increase the effectiveness of common medication-based strategies for these conditions or reduce their adverse effects [14, 15].

In this review, we provide a brief overview of the role of gut dysbiosis in various CNS disorders, and the influence of different CNS medications on gut dysbiosis and how their bioavailability and actions could be affected by the patient’s GM. We also discuss the contribution of GM modulation using diet, antibiotics, fecal microbiota transplantation (FMT), prebiotics, probiotics, and microbial trimethylamine (TMA)-lyase inhibitors to the management of CNS disorders. Various illnesses affecting CNS can be better diagnosed, prevented, and managed by targeting the gut-brain axis.

Unlike the previously published reviews, this work provides holistic and application-oriented approach by addressing the bidirectional interactions between GM and CNS pharmacological agents and by incorporating the influence of microbiota-based therapies alone or in combination with traditional CNS pharmacological agents. Importantly, assessing the composition of GM, interactions among different host factors, and outcomes of pharmacological agents will be important to secure the success of this strategy in the management of various CNS disorders.

## Gut Dysbiosis and Various CNS Disorders

**Fig. 1:**
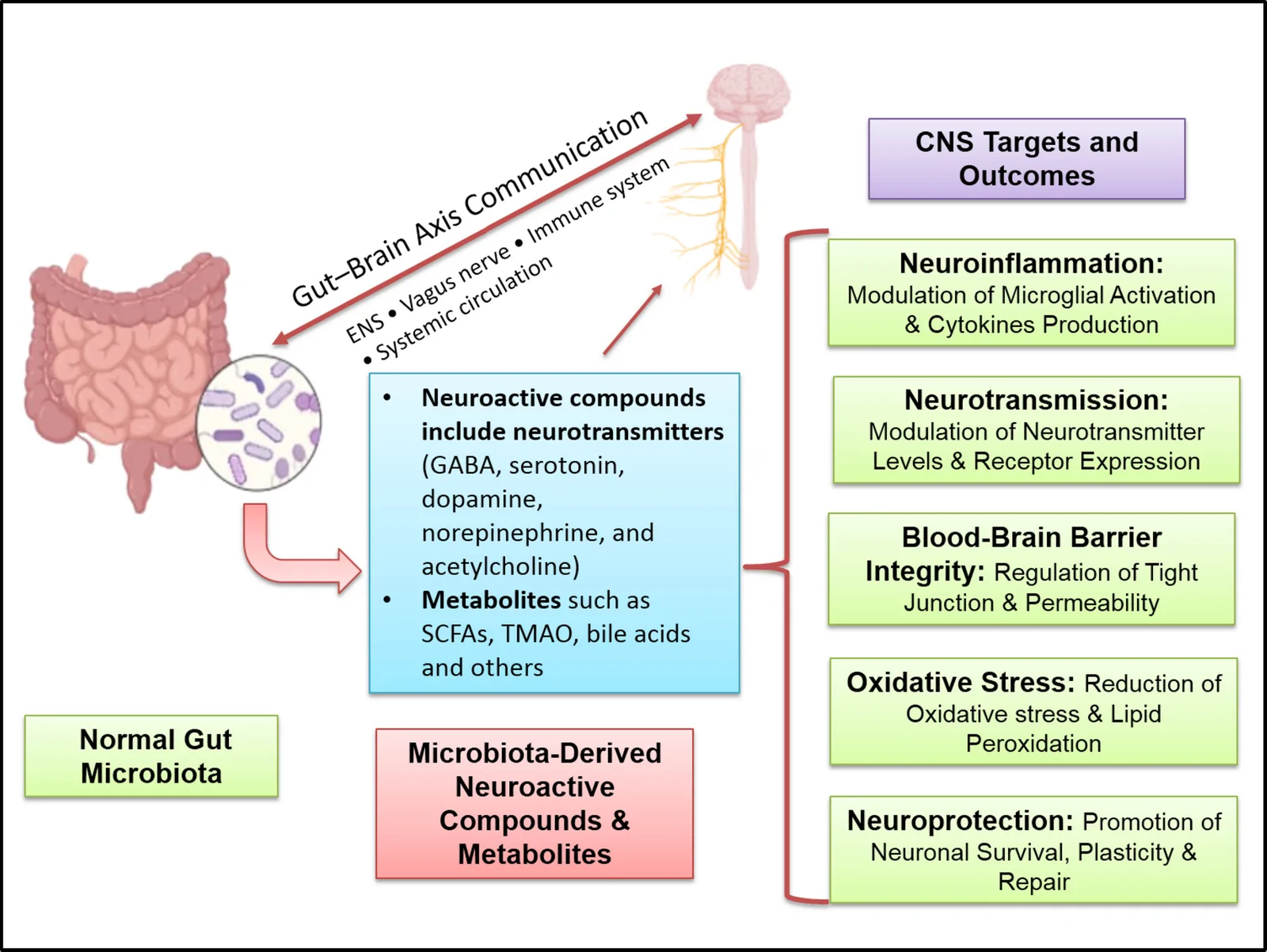
Schematic representation of the main mechanisms influencing gut microbiota (GM)–brain communication. Gut microorganisms affect the generation of a wide range of neuroactive compounds, including γ-aminobutyric acid (GABA), serotonin, dopamine, norepinephrine, and acetylcholine, as well as metabolites such as short-chain fatty acids (SCFAs), trimethylamine N-oxide (TMAO), bile acids, and other microbial products. These microbiota-derived molecules affect the function of central nervous system (CNS) through several interconnected pathways, like signaling through the vagus nerve, the enteric nervous system (ENS), the immune system, and the systemic circulation. Collectively, GM plays a central role in the bidirectional gut–brain axis communication to regulate the neurotransmission, the integrity of blood–brain barrier, the neuroinflammation, and thus the CNS and behavioral functions

In the recent years, an increasing body of evidence has highlighted the role of GM as a critical driver in the pathogenesis of several neurodevelopmental or neurodegenerative disorders via the microbiota–gut–brain axis; however, the robustness of this evidence differs across various disorders [16]. As shown in Fig. 1, enteric nervous system (ENS), vagus nerve, immune system, and metabolites generated by microbes in the gut, like short-chain fatty acids (SCFAs), are some examples of the mechanisms by which brain and intestine interact [17, 18]. Moreover, the ability of certain microbiota to synthesize several neurotransmitters, including gamma-aminobutyric acid (GABA) [19], serotonin [20], noradrenaline [21], acetylcholine [22], histamine [22], and dopamine [22], has obviously been reported. Among these neuroactive substances, GABA, serotonin, and tryptophan metabolites do not influence directly the CNS as they are not able to cross blood–brain barrier but they can exert their effects through interaction with cells of ENS [23].

The metabolites or endotoxins released by the GM can significantly affect neurotransmitters levels and their precursors as well as the expression of their related receptors in the CNS via vagus nerve pathway or released products from gut into the systemic blood circulation, thereby affecting cognitive behavior and other brain functions [24]. However, understanding whether these bacterial byproducts or metabolites achieve enough high concentrations to exert certain physiological or pharmacological action needs to be investigated in detail in future studies. Thus, the revealed linkages across neurological, endocrine, and immunological systems which include the GM have significantly increased interest in investigating the gut-brain axis [25].

GM can affect brain health through influencing metabolic and inflammatory pathways that affect vulnerability to neurological diseases. However, the reported microbiota changes may reflect shared metabolic and immune perturbations rather than specific disease signatures [26]. Dysbiosis is strongly correlated not only with intestinal disorders such as irritable bowel syndrome, celiac disease, inflammatory bowel disease, and metabolic disorders like diabetes mellitus and obesity but also with neurological diseases [27, 28]. Hence, imbalances in the digestive tract or the CNS may impact each other’s homeostasis.

### Neurodegenerative Diseases

Neurodegenerative diseases, which can impact the vital activities in the CNS, are closely correlated with symptoms of persistent gut dysfunctions [29]. Multiple studies have demonstrated that gut-brain connecting neurons mediate the interactions between CNS and gut functions, influencing the development and progression of neurodegenerative diseases [29, 30]. The disruption of the intestinal epithelial barrier and the resulting local and systemic inflammatory states are both caused by gut dysbiosis [31]. Additionally, dysbiosis is correlated with increase in brain parenchymal permeability, neuroinflammation, and malfunction of neuronal cells [32].

Importantly, brains of patients with AD have shown high concentrations of gram-negative bacterial compounds, such as lipopolysaccharide (LPS). The presence of amyloid-beta (Aβ) deposits in the plaques together with the elevated levels of LPS indicate potential interactions between the two elements [33]. Furthermore, LPS may enhance microglial activation and a variety of cognitive dysfunctions via activating nuclear factor kappa B (NF-κB) signaling at the neuronal level [34]. In addition, LPS enhances the absorption of Aβ across blood–brain barrier, which impacts its transport [35]. Endogenous Aβ generation may include peripheral source, like the gut, with a significant impact on CNS pathology; however, the exact mechanism of transport between the periphery (e.g., gut) and the brain remains to be investigated in future studies, with some speculated pathways like crossing of blood–brain barrier through receptor-mediated transport or via cargo within immune [36]. In AD, there are elevated levels of Aβ in the intestinal mucosa both in experimental models [37] and in patients [38]. When the intestinal epithelium and blood–brain barrier are damaged in a dysbiotic environment, amyloid peptides can enter the bloodstream and clump together in the CNS [39]. Notably, neuronal survival, development, and synaptic plasticity are all greatly affected by brain-derived neurotrophic factor (BDNF), whose expression is inhibited in the presence of dysbiosis [40].

Specifically, Vogt et al. revealed significant reduction of levels of *Firmicutes* and *Bifidobacterium*, and an elevation of the level of *Bacteroidetes* in patients with AD compared to healthy individuals [41]. These outcomes were confirmed by Liu et al. [42] whereas Zhuang et al. demonstrated a significant decrease in the abundance of *Bacteroidetes* and increase in the abundance of *Enterococcaceae*, *Ruminococcaceae*, and *Lactobacillus* in AD patients [43]. These discrepancies among various studies could be due to difference in illness severity, diagnostic criteria, lifestyle and race factors, presence of comorbidities, and use of medications [44]. Thus, well-designed and standardized studies are required in the future for precise evaluation of GM changes in AD.

An increasing body of evidence suggests that gut dysbiosis can modulate the progression of other neurodegenerative illnesses, including PD. PD is frequently accompanied by changes in ENS and gastrointestinal symptoms [45]. Reliable data demonstrates that dysbiosis can contribute to progression of neurodegeneration in PD patients, with increase in abundance specific bacterial families (e.g., *Akkermansiaceae*) and decrease in microbiota that generates beneficial SCFAs [46]. Moreover, PD patients possess decreased levels of beneficial bacterial families, such as *Lachnospiraceae* and *Lactobacillaceae* as compared with healthy individuals. There are several metabolic pathways that are also affected by PD-related dysbiosis, including those responsible for metabolism of amino acids, carbohydrates, and xenobiotics [47, 48].

According to a previous study, α-synuclein (α-Syn)-mutant mice showed dopaminergic neurotoxicity, worsened neuropathology, and neuromotor deficits which were correlated with mild chronic intestinal inflammation [49]. Research has demonstrated that α-syn pathologically aggregates in the intestines of PD patients, similar to what occurs in AD [50]. It is worth noting that abnormal α-syn could be identified in the initial phases of PD, which can be 10–20 years before the appearance of significant symptoms [51, 52]. The severity of α-syn aggregation correlates with the levels of constipation and the extent of enteric neuronal loss in PD patients [53]. Interestingly, there is an experimental evidence on how α-syn precipitates injected into the intestinal wall could be transported to the brain by the vagus nerve, leading to neuropathology similar to PD and its associated motor abnormalities [54, 55].

Although GM changes are recognized in PD, these observations may be affected by presence of other factors, especially age [56, 57]. Common changes are observed in PD and aging like increase in the abundance of *Alistipes* and *Akkermansia* and decrease in the abundance of *Blautia* and *Faecalibacterium*. These common microbiome signatures may influence the outcomes of studies [56] where differentiating microbial changes related to PD rather than aging may be required for prevention of the evaluated overlap in microbial signatures along with disease-related functional differences [57].

In an ALS experimental model, the onset of movement disorders is also preceded by changes in SCFA-producing bacteria (e.g., *Akkermansiaceae*, *Prevotellaceae*, and *Oscillospiraceae*) and prokaryota families (e.g., *Bacteroidiaceae*, *Lactobacillaceae*, and *Bifidobacteriaceae*) [58]. Moreover, ALS patients tend to have lower levels of helpful *prokaryota* families, such as *Peptostreptococcaceae*. Obviously, this illness phenotype is worsened by the loss of certain microbiota due to environmental changes or antibiotic usage [59]. It is worthy to note that the most frequent genetic mutation that contributes to ALS is C9orf72, where the microbiome is enriched with pro-inflammatory bacteria, leading to increased systemic and neuronal inflammation in C9orf72 knockout mice [60].

### Depression

Major depressive disorder (MDD) is correlated with an elevated suicide rate and other devastating consequences [61]. Gut dysbiosis is linked to the onset and development of depression [62]. In individuals with MDD, differences in the abundance of 16 bacterial families and variations in the composition of the fecal microbiota across four phyla have been observed [63]. Additional research revealed that compared to healthy persons, those with MDD show decreased levels of *Bacteroidetes *and greater amounts of *Prevotella*, *Klebsiella*, *Streptococcus*, and *Clostridium XI* [64]. Furthermore, premenopausal women suffering from depression exhibit elevated levels of *Klebsiella aerogenes*, a bacteria capable of degrading estradiol compared to control patients [65].

Interestingly, germ-free mice could exhibit depressive-like behaviors after receiving fecal microbial transplants from depressed people [66]. Alternatively, by drastically reducing the number of gut microbial species that potentially regulate the host’s immunological and metabolic activity, FMT from healthy Sprague–Dawley rats could prevent the onset of depression in Fawn-hooded rats [67]. Thus, GM dysbiosis may play a pivotal role in the pathophysiology of depression through affecting the expression of proteins or factors involved in gut-brain axis [5].

Notably, GM composition can be reshaped by several dietary, environmental, and other host-related factors, including medications and lifestyle habits [68, 69]. The diversity of these factors complicates the exact identification of specific microbial taxa that are correlated with depression [69]. Antidepressants may significantly affect diversity of GM, with observed variations in β-diversity between individuals not exposed and those exposed to antidepressants [70] and so exposure to antidepressants may influence interpretation to GM changes in patients with depression. Different classes of antidepressants in addition to various lifestyle habits such as sleep patterns, physical activity, and dietary factors may all impact the inter-individual variations in the composition of GM reported in patients with depression [68]. Taken as a whole, GM composition is susceptible to variations in patients with depression and may represent a new avenue of research for treatment and prevention of this neurological illness.

### Schizophrenia

One percent of the global population suffers from schizophrenia where patients suffering from this disorder display both positive and negative symptoms, including hallucinations, disorganized speech, lack of interest and motivation, and cognitive deficiencies such as impaired memory and executive functioning [71]. A cross-sectional investigation demonstrated that certain gut bacteria, like *Haemophilus*, were either absent or significantly reduced in schizophrenic patients compared to healthy controls, suggesting gut dysbiosis [72]. Another study revealed that there is a favorable link between the amount of *Haemophilus* and the absence of schizophrenia symptoms [73]. Possible explanations for these conflicting results may be attributed to difference in disease severity or subtypes, antipsychotic medications, and dietary factors, as well as variability in study design and methods of microbial analyses [73, 74]. Moreover, the methodological diversity and relatively small sample sizes may limit generalizability and reproducibility of the observed outcomes, highlighting the requirement of standardized and large multicenter clinical studies to confirm schizophrenia-related microbial signatures [75].

Concerning other studies revealing GM changes in schizophrenia, these patients have demonstrated increased abundance of *Lachnospiraceae* and *Veillonella* and decreased amount of *Ruminococcus* and *Roseburia* [76, 77]. Schizophrenia patients have also shown to possess facultative anaerobes, such as *Lactobacillus fermentum* and *Enterococcus faecium*, which are extremely unusual in healthy people [78]. The severity of schizophrenia symptoms has been positively correlated with *Succinivibrio* abundance and negatively correlated with *Corynebacterium* abundance [79]. In addition, gut dysbiosis can modify the glutamate-glutamine-GABA cycle, a neurochemical pathway that is implicated in the pathogenesis of schizophrenia, and thus altering neurological function and neurochemical levels in these patients [80, 81]. As a result, schizophrenia patients may benefit from a supplement of beneficial microbiota, which is either not present or significantly reduced in individuals with this disorder, in addition to its favorable outcomes on gut dysbiosis.

### Epilepsy

The hallmark of epilepsy, a chronic neurological condition, is seizures that happen suddenly and without warning [82]. The exact etiology of epilepsy remains a mystery in nearly half of patients. Intriguingly, some epileptic patients have shown gut dysbiosis; hence, restoring GM may alleviate epilepsy and increase the effectiveness of anticonvulsant medications, particularly in those with drug-resistant epilepsy [83].

Notably, animals under stress suffer from gut flora changes, making them more susceptible to epileptic seizures [84]. These results were confirmed by subjecting non-stressed rats to fecal content transplantation from stressed rats before induction of epilepsy [85]. In addition, pharmacologically induced seizures are more likely to occur in patients with inflammatory bowel disease [86]. Another study assessed the correlation between changes in GM and increase in seizure vulnerability where intestinal infection with gram negative bacteria, such as bacterium *B. fragilis* or LPS, caused cerebral cavernous malformations and structural abnormalities in brain capillaries, predisposing more the genetically susceptible mice to stroke and seizures [87].

In accordance with experimental studies, patients with refractory epilepsy show greater variations of microbiota composition compared to those responding well to anti-epileptic medications [6, 88]. When compared to control samples, children with refractory epilepsy revealed microbiota that were more abundant with *Firmicutes* and *Actinobacteria* and less abundant with *Bacteroidetes* and *Proteobacteria*, according to taxonomic analysis [89]. Overall, these studies reveal a correlation between GM composition and susceptibility to epileptic activity or seizure.

Emerging evidence also reveals that ketogenic diet, a diet including high fat, low carbohydrate, and adequate protein, can modify the microbiota-gut-brain axis with impact on the promising clinical response reported during management of drug-resistant epilepsy [90, 91]. Notably, current bioinformatics and metagenomic analyses have revealed a significant increase in the prominence of specific microbial taxa, including *Parabacteroides gordonii* and *Akkermansia muciniphila* which have been previously evaluated as putative antiepileptic species [92], as an outcome of intervention with ketogenic diet. These taxa are well known for their capabilities to enhance metabolic lipid utilization in vitro [93]. However, other data suggests that change in microbiota composition and its relation to ketogenic diet could be caused by non-nutritional factors such as physical activity, age, pre-existing disease, and use of medications [94, 95]. These factors may also be partly responsible for the observed discrepancies between adult and pediatric microbiome-related studies in epilepsy [96].

### Anxiety

Anxiety is one of the prevalent mental health conditions that is caused by several psychological, biological, and social factors [97]. Being socially isolated is one of the contributing risk factor that elevates susceptibility to anxiety, where patients with anxiety usually suffer from poor social skills, which can further aggravate social withdrawal [98]. A decline in the fraction of *Firmicutes* to *Bacteroidetes* and an upregulation of *Prevotella* abundance were observed in people who were socially excluded [99]. Patients suffering from generalized anxiety disorder also showed decreased levels of *Firmicutes* spp. and poorer microbial diversity and richness along with increased concentrations of *Fusobacteria* and *Bacteroidetes* when compared with healthy persons [100]. Mice with higher anxiety levels also showed considerably lower amounts of *Firmicutes* [101], confirming that fecal microbiome could impact anxiety-related behavior [99].

In brief, anxiety can have a profound effect on the diversity and richness of microbes where people suffering from this condition have increased abundance of *Bacteroidetes* and *Fusobacteria* and decreased abundance of *Firmicutes* [100]. Several genera of gram-negative bacteria, including *Prevotella*, *Lactobacillales*, *Sellimonas*, *Streptococcus*, and *Enterococcus*, are positively correlated with anxiety [5]. Functional alterations in GM may directly affect anxiety pathophysiology, including distinctive changes in microbial related metabolite production, like SCFAs (e.g., acetate, butyrate, and propionate) and neurotransmitter-related compounds (e.g., tryptophan metabolites and GABA), affecting gut–brain axis and modulating immune–inflammatory signaling [102]. It is worthy to note that several microbial species within the same genera can have distinct roles or even opposite effects on anxiety, so targeting these variations in GM could be a reliable approach for prevention of anxiety [5].

## GM Regulations in Different CNS Disorders

**Fig. 2:**
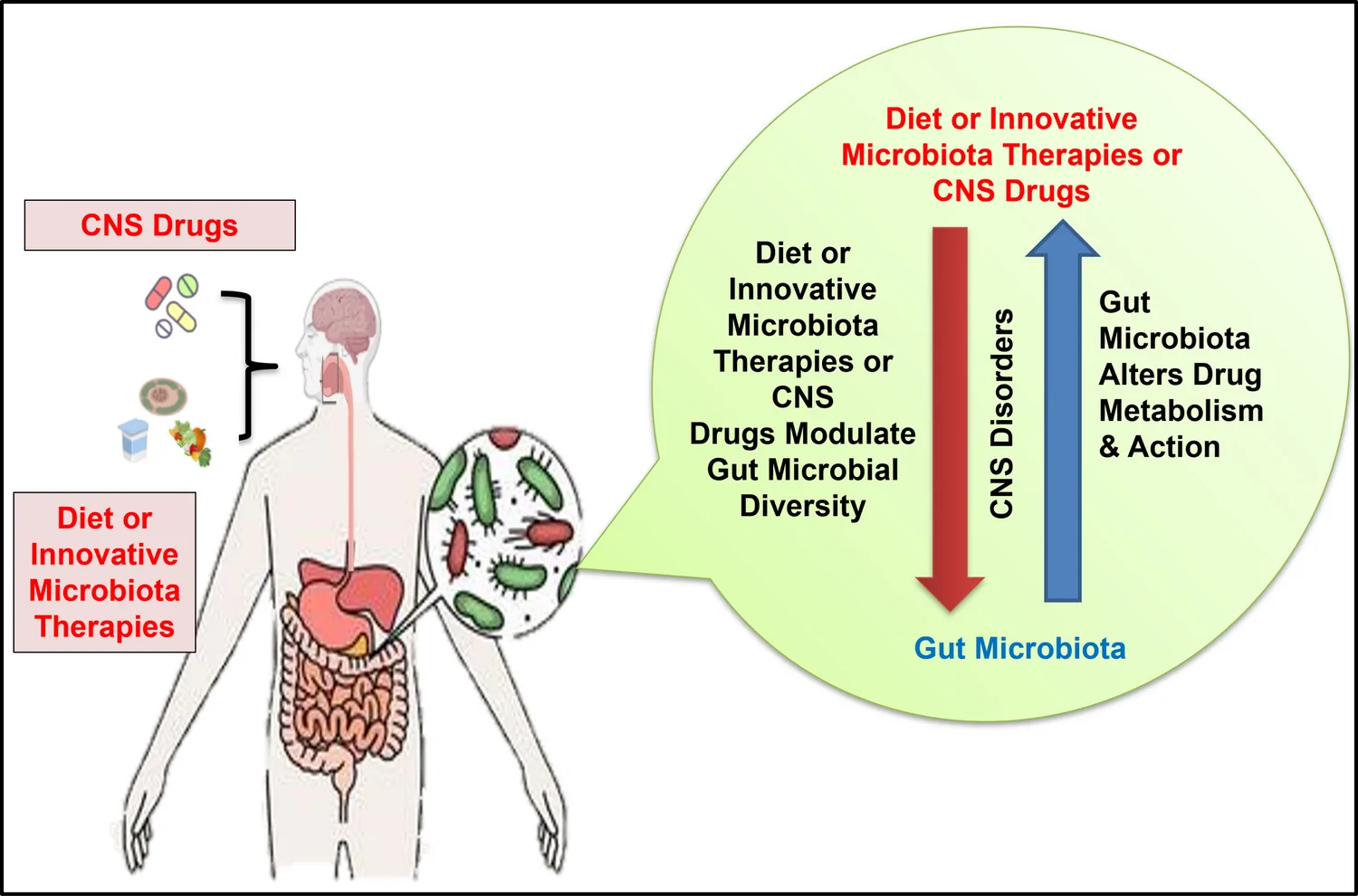
Schematic representation of the bidirectional interactions among diet, innovative microbiota therapies, central nervous system (CNS) acting drugs, and gut microbiota (GM) in the management of CNS disorders. Dietary interventions, innovative microbiota therapies, and CNS-acting pharmacological agents can alter gut microbial composition and diversity. In turn, GM can affect metabolism, bioavailability, efficacy, and adverse effects of CNS acting drugs. These reciprocal interactions may influence disease progression and therapeutic outcomes through the microbiota–gut–brain axis. The red downward arrow indicates the modulatory effects of diet, microbiota-targeted therapies, and CNS acting drugs on gut microbial diversity, whereas the blue upward arrow represents the influence of GM on drug metabolism and its pharmacological action

Gut–brain axis is a main regulator of human physiology where dysbiosis is linked to pathogenesis of various diseases which affect the gut, the systemic health, and the brain. The proposed mechanisms include altered neural or vagal signaling, disrupted microbial-related metabolites (SCFAs, tryptophan derivatives, and trimethylamine N-oxide (TMAO)), and modulated immune system contributing to neuroinflammation [103]. Dietary interventions, prebiotics, probiotics, antibiotics, and FMT have all shown promising outcomes in reversing gut dysbiosis altogether with beneficial effects in the management of CNS-related disorders. The interplay of dietary approaches, innovative microbiota therapies, and pharmacological agents in the management of CNS-related disorders are shown in Fig. 2.

### Dietary Interventions

Factors such as food, environment, age, and medicine can all have impacts on reshaping of GM [104]. Dietary approaches could affect mental activities and brain health including synaptic plasticity where various gut hormones can enter the brain, controlling the cognitive ability [105, 106]. A person’s nutritional intake can also influence neurotransmitter levels, synaptic transmission, cognitive capacities, membrane fluidity, and signal transduction pathways [107]. Accordingly, dietary factors can significantly impact the microbiota-brain connection, which in turn affects mood, cognitive function, and even the development of neurodegenerative diseases through influencing the brain health [108].

The effects of Western and Mediterranean diets on GM and CNS functions are different where both have been subjected to tremendous research. Research has linked the Western diet, which is known for abundance of processed foods, sugar, bad fats, and minimal fiber, to negative impacts on gut health and brain function [109]. Elevated neuroinflammation and poor brain health are symptoms of dysbiosis, which occurs when the microflora in the gut is disrupted by this eating pattern, encouraging the growth of harmful bacteria. A Western diet may hasten cognitive decline and increase vulnerability to neurodegenerative disorders including AD and PD [110]. Dysbiosis caused by this dietary approach leads to an imbalance in gut health, including overgrowth of inflammation-causing *Firmicutes* bacteria and decrease in *Bacteroidetes*, the bacteria responsible for fiber fermentation and SCFA production [111].

The production of SCFAs, which are crucial for maintaining a healthy intestinal barrier and controlling immunological responses, is negatively affected by low-fiber diets [112]. When an imbalance occurs between pathogenic and beneficial bacteria in the gut, a condition known as “leaky gut” develops, making it easier for toxic byproducts of bacteria like LPS to enter the circulation [113]. Neuroinflammation and neuronal injury can occur in the brain as a result of accumulation of these microbial compounds causing systemic inflammation [114]. Collectively, cognitive decline and brain dysfunction are worsened by microbial dysbiosis, which is caused by poor diets that are high in fat and lack fiber [115].

A healthy diet, on the other hand, can boost cognitive function and lower the risk of neurodegenerative illnesses through maintenance of a balanced microbiota [116]. The Mediterranean diet, which is high in plant-based foods (e.g., fruits and vegetables, whole grains, legumes), healthy fats (mostly olive oil), and lean proteins like fish [117], improves cognitive performance and lowers the incidence of neurodegenerative disorders [118]. The prevalence of good bacteria as a consequence of this dietary approach increases the production of metabolites such SCFAs, which have anti-inflammatory and neuroprotective effects [119]. Polyphenols, antioxidants, and healthy fats that are the main components of the Mediterranean diet lower inflammation and oxidative stress in CNS, which in turn improve cognitive health. Researchers have found that people who follow Mediterranean diet show less cognitive deterioration and higher overall cognitive ability as they get older compared to those who do not follow this diet [120]. Therefore, to improve cognitive performance and protect the brain from age-related neurodegenerative illnesses, it is essential to follow a dietary approach that supports beneficial gut bacteria and their anti-inflammatory activities [121].

Crucially, rise in rate of depression and other mental illnesses may be attributed to decline in omega-3 fatty acid consumption [9, 122]. Cognitive decline is caused by decrease in brain docosahexaenoic acid (DHA), which is linked to number of CNS illnesses, including attention deficit hyperactivity disorder, depression, cognitive impairment, AD, dyslexia, and schizophrenia [123–125]. Omega-3 polyunsaturated fatty acids, such as DHA and eicosapentaenoic acid (EPA), are abundant in fish where reducing neuroinflammation is one of crucial roles played by DHA and EPA on brain function and neurocognitive development [126]. Eating a diet rich in omega-3 fatty acids improves brain function through positively regulating the genes that are involved in synaptic activities [127]. These findings highlight the importance of dietary interventions in supporting both gut health and brain functions.

### Prebiotics

Prebiotics are indigestible food components that aid the growth and activities of various bacteria in gut with positive effects on the host [128]. These components include fructooligosaccharides, xylooligosaccharides, and galactooligosaccharides. The functional food known as prebiotics has a distinctive advantage over probiotics as it encourages the growth of numerous beneficial bacteria rather than just one type of germ [129]. Neuroprotection is also greatly supported by prebiotics which are dietary components that are resistant to digestion [130]. Fermentation of dietary fibers by gut bacteria produces SCFAs like butyrate, which is known to have many positive impacts on brain function. The neuroprotective benefits of butyrate are attributed to its ability to decrease neuroinflammation, improve mitochondrial activity, and facilitate the elimination of harmful substances such as Aβ [131].

In general, prebiotics have been reported to enhance brain function and prevent neurological related illnesses such as AD [132], dementia [133], irritable bowel syndrome [134], PD [135], and autism spectrum disorder [136]. Fructo- and galactooligosaccharide therapies have shown favorable effects on chronically stressed rats through improving depression and anxiety-like behaviors via targeting the microbiota-gut-brain axis [137]. Researchers have also shown that prebiotics could raise levels of cecal SCFAs (acetate and propionate) while decreasing levels of corticosterone and chronic stress-induced proinflammatory cytokines in experimental animals [137, 138]. Similarly, treatment with prebiotic like Bimuno-galactooligosaccharides improved cognitive functions and significantly reduced microglia activation and the expression of inducible nitric oxide synthase, CD68, CD32, suppressor of cytokine signaling 3, and interleukin-6 (IL-6) in mice together with enhancement of beta diversity, *Bifidobacterium* proliferation, and other anti-inflammatory microorganisms [139].

Importantly, one of the prebiotics that are derived from plant, bacterial, or yeast cell walls is mannooligosaccharides (MOS) [140]. MOS has been reported to lessen systemic inflammation and boost the immune system with rise of *Lactobacillus* and promotion of butyrate synthesis [141, 142]. In mice supplemented with MOS (0.12% w/v in drinking water), better cognitive function, less anxiety, and less obsessive–compulsive behaviors were observed compared to untreated animals through control of build-up of Aβ protein, redox status, and neuroinflammatory response in the brain system [141, 142]. Another prebiotic that has shown promising results as a treatment for PD is polymannuronic acid. Experimentally, oral administration of polymannuronic acid significantly enhanced tyrosine hydroxylase gene expression and decreased dopaminergic neuron loss in the striatum through promoting SCFAs-mediated anti-inflammatory and anti-apoptotic actions [143]. Applying oligofructose-enriched inulin also raised serum butyrate in patients with schizophrenia [144]. Furthermore, alterations of fecal flora and subsequent improvements of gastrointestinal and psychotic symptoms were observed in patients treated with another prebiotic like lactosucrose [145]. Despite the reported positive impacts of prebiotics, some limitations are present in their use including significant inter-individual variability in response and heterogeneity in prebiotic type, dosage, and intervention duration in different studies. Additionally, a dose–response relationship of prebiotic should be evaluated to select the minimum effective dose and the targeted outcome (e.g., selected bacteria genus or species) to be tested in subsequent studies before using this supplementation with presumed prebiotic effect [146]. The variations of these previously mentioned factors could reduce the strength of conclusion of performed studies and limit the reproducibility of the findings upon clinical application [147–149].

### Probiotics

Probiotics, which are live beneficial bacteria, are important in supporting gut health and controlling of gut–brain axis [150]. Evidence suggests that some types of probiotics like *Bifidobacterium* and *Lactobacillus* species may provide direct protection to the nervous system. In addition to influencing the production of neuroactive chemicals like 5-HT, these strains can modulate the immune system and decrease inflammation of the gut [151]. Improving gut health, lowering systemic inflammation, and preserving the blood–brain barrier integrity are all potential consequences of probiotics use and their positive outcomes on cognitive function and neuronal functions [152].

Through colonizing with the host and modifying the composition of GM, probiotics can have positive impacts on the host. Probiotics can control both the mucosal and the systemic immune functions with improvement of nutrient absorption and gut health [153]. Fermented dairy products and fecal microbiome have traditionally been used as main sources of probiotics like *Lactobacilli*, *Bifidobacteria*, and other lactic acid-producing bacteria [154]. By releasing antimicrobial compounds like organic acids, probiotics can decrease endotoxemia, minimize intestinal permeability, and suppress inflammatory response [155]. Probiotics outcompete enteropathogens like *Salmonella typhi*, *Helicobacter pylori*, and *Entamoeba histolytica* by promoting the fermentation of substrates that produce a wide variety of organic acids and volatile fatty acids, thus lowering the pH level in the gut [156]. Probiotics can influence the ENS through controlling the composition of GM and their generated metabolites and, hence, affecting CNS functions. As an example, *Lactobacillus reuteri* has shown an effect on the gut-brain axis by specifically raising the excitability of colonic post-hyperpolarization neurons, a kind of ENS neuron which influences the transmission of sensory information to the CNS [157].

Prolonged supplementation with probiotic, containing *Bifidobacterium animalis* subsp. *lactis* CP-9 and *Bifidobacterium longum* subsp. *infantis* BLI-02, and *Bifidobacterium breve* Bv-889, has revealed to lower the production of inflammatory cytokines, improve the antioxidant status, and increase the serum BDNF levels in AD patients. These results suggest that probiotics can help symptomatic AD patients delay the deterioration of cognitive function [158]. Additionally, SLAB51 probiotics, which include *Bifidobacteria*, *Streptococcus thermophilus*, and *Lactobacillus*, can reduce Aβ protein deposition in mice with AD through restoring autophagic function, inhibiting systemic inflammation, and reversing the damaged ubiquitin proteasome system [159, 160].

Probiotics Bifico, which is a mixture of *Bifidobacterium*, *Lactobacillus acidophilus*, and *Enterococcus faecalis*, were found to improve the symptoms of depression and anxiety in ovariectomized rats. This was achieved by lowering inflammation in the gut through improving the general microbiome, which in turn reduced neuroinflammation in brain by inhibiting the NOD-like receptor protein 3 (NLRP3) inflammasome signaling pathways [161]. By controlling the composition of GM and the metabolism of tryptophan, *Bifidobacterium breve* CCFM1025 was also revealed to alleviate the major depressive illness [162]. In another study, a mixture of probiotic strains was investigated, including *Lactobacillus plantarum* (DSM32244), *Lactobacillus acidophilus* (DSM32241), *Lactobacillus helveticus* (DSM32242), *Lactobacillus brevis* (DSM11988), *Lactobacillus casei* (DSM32243), *Bifidobacterium lactis* (DSM32246), and *lactis* DSM32247. After 4 months of treatment with this probiotic mixture in patients with drug-resistant epilepsy, 28.9% of these patients had a 50% reduction in seizure frequency, which was promising [163]. In addition, the beneficial impact of *Bifidobacterium* was highlighted in another research for the control of schizophrenia-related symptoms of anxiety and depression [164].

Although probiotics are generally accepted as safe intervention, immunocompromised patients and critically ill elderly people are at increased risk of severe adverse effects including sepsis from taking excessive probiotics [165]. Another concern has been suggested concerning their possible interaction with serotonergic pathways, with a hypothesized risk of serotonin syndrome when used together with selective serotonin reuptake inhibitors (SSRIs) in patients with depression [166, 167]. There is no supportive evidence which confirms this hypothesis; however, caution should be undertaken upon prolonged use of probiotics specifically in elderly patients with other chronic neurological disorders. Furthermore, there is a challenge in selecting appropriate strain of probiotic or mixtures of strains when evaluating their safety or efficacy or upon their selection for targeting specific disease in certain patients [168].

### FMT

The goal of FMT is to alleviate disease symptoms by re-establishing a healthy GM by transferring stable microbial communities from healthy donors’ stool to patients with particular disease [169]. With a success rate of up to 90%, the FMT approach was initially employed in 1958 to treat refractory and recurring *Clostridium difficile* infections, thereby being regarded an alternative to antibiotics [170]. Although FMT is widely accepted as a safe process, some adverse effects may be linked to this procedure including nausea, bloating, abdominal discomfort, and diarrhea [171]. Studies also showed that FMT is generally safe and effective in mildly or moderately immunocompromised patients [172, 173].

The effects of FMT have been assessed in various models of CNS injury. Transplanting the GM of healthy mice was found to reduce neuroinflammation caused by spinal cord injury and improve recovery rates by re-establishing tight junction ability of the intestinal epithelium together with increase in the production of microbial SCFAs [174]. FMT from healthy C57BL/6 J mice to mice with PD revealed also the potential to improve gut dysbiosis and PD symptoms by increasing the abundance of *Clostridiales* and *Firmicutes* and decreasing the abundance of *Proteobacteria*, *Turicibacterales*, and *Enterobacteriales* [175]. Experimental evidence suggests that FMT can reduce PD symptoms by restoring SCFAs to their baseline levels [176]. FMT has the ability to reduce astrocyte and microglia activation in the substantia nigra, as well as inhibit the inflammatory signaling cascade involving toll-like receptor 4 (TLR4) and tumor necrosis factor alpha (TNF-α), which in turn reduce the development of PD [177]. In patients with mild to moderate PD, extended FMT therapy seems to be a safe and an alternative option which reduces daily motor and non-motor symptoms over the course of treatment [178].

Experimentally, FMT from healthy mice (wild-type) could also improve cognitive deficits and Aβ plaques in AD mice, in the absence of antibiotic use [179]. By modifying the composition of GM and inhibiting the LPS-TLR4 pathway, FMT effectively could reduce cognitive deficits through improvement of intestinal and neuroinflammation and reduction of Aβ formation [180]. The cognitive impairment and pathological changes observed in FAD^4T^ mice, a genetically modified mouse model of AD, could be reduced through frequent FMT via lowering Aβ and tau pathology and neuroinflammation. This provides a new avenue about the interconnection between microbiota-gut-brain axis and AD by re-establishing the GM balance [181]. Through inhibiting glial activation and NLRP3 inflammasomes in the brain, FMT could improve stress-induced depressive-like behaviors in rats [182]. In addition, FMT was observed to ameliorate autism spectrum disorder in mice produced by valproic acid by influencing the synaptic serotonergic and glutamatergic signaling pathways [183].

Among the most important considerations in FMT are the age of the donor and the severity of the condition. Yet, FMT’s safety profile and good efficacy have led to its extensive usage in the management of neurological disorders, including autism and epilepsy [184, 185]. Some of the minor side effects of FMT, being a biological therapy, include constipation, diarrhea, stomach pain, and temporary low fever that may go away in a few days [186]. Importantly, regulatory frameworks and ethical considerations should be followed regarding the informed consent, the methods of screening of donor samples, and the accessibility to data or outcomes of trials [187]. Certain regulations should be also considered before use of FMT to minimize variability in results or outcomes including precise evaluation of its active components and mechanisms of action in various disease contexts in addition to utilization of standardized procedures for donor selection, sample preparation and administration, and long-term safety assessment [188, 189] to reduce inconsistency in outcomes of clinical trials [189].

### Antibiotics

Clinical practice frequently involves the use of antibiotics to treat bacterial illnesses. In animal and human research, antibiotic use is usually correlated with intestine ecological abnormalities together with CNS dysfunction [190, 191]. Long-lasting changes in GM due to use of antibiotics can impact the systemic levels of neuromodulators like tryptophan and kynurenine, affecting BDNF expression in the hippocampus, and ultimately increasing the risk of anxiety and cognitive decline [192]. For instance, ampicillin could raise serum corticosterone level experimentally, which in turn led to anxious behaviors in rats and decreased spatial memory [193]. Intestinal ecological problems can be induced together with inhibition of tight junction protein expression by oral administration of vancomycin and ampicillin, leading to increased leakage of LPS into the bloodstream. The entry of LPS into the brain, particularly the hippocampal region, enhances neuroinflammation and affects cognitive function [194] via activation of microglia to stimulate NF-κB production and promote the release of inflammatory cytokines [195]. Also, enrofloxacin revealed a decrease in the *Firmicutes*/*Bacteroidetes* ratio, which might be linked to gut dysbiosis and lead to anxiety-like behaviors in zebrafish through altering brain peptides by disrupting the microbiota-gut-brain axis and the synthesis of neurotransmitters [196].

Conversely, by reducing systemic inflammation, vancomycin pretreatment protected neurons in mice with 1-methyl-4-phenyl-1, 2, 3, 6-tetrahydropyridine-induced PD [197]. Another study demonstrated that rifaximin protected transgenic PD mice’s brains by influencing the gut-brain axis and GM [198]. Experimental evidence also suggests that minocycline therapy could alleviate anxiety-like behaviors by lowering IL-6 and TNF-α levels [199]. Furthermore, by blocking the release of high mobility group box 1 in neurons and microglia, minocycline considerably improved moderate stress-induced cognitive and behavioral impairments [200]. Additionally, ciprofloxacin and metronidazole, when used for short periods of time, were shown to raise 5-HT levels, which could alleviate symptoms of anxiety and depression [201]. Importantly, weight increase, uterine fat deposition, and macrophage infiltration in adipose tissues were experimentally observed side effects of olanzapine, which were mitigated by GM modulation using an antibiotic cocktail consisting of neomycin, metronidazole, and polymyxin B [202].

On the other hand, antibiotic use can affect GM homeostasis, allowing the expansion and growth of resistant strains and assisting the rapid transfer and dissemination of resistance genes to antibiotics within the gut ecosystem through various mechanisms like conjugation, transformation, and transduction, identifying one of the major risks related to prolonged use of antibiotics [203]. Moreover, antibiotics can significantly modify microbial metabolites, like secondary bile acids and beneficial SCFAs which are essential for preserving gut epithelial integrity [204, 205]. Furthermore, antibiotic-induced depletion of certain microbiota can influence homeostasis of immune system, leading to elevated susceptibility to viral and bacterial infections [203]. Collectively, despite their beneficial effects, these findings emphasize the importance of judicious use of antibiotics as they may have potential long-lasting outcomes on composition of GM, regulation of immune system, and dissemination of antimicrobial resistance.

### Microbial TMA-Lyase Inhibitors

Choline, betaine, and L-carnitine are dietary elements that can be converted to TMA by a diverse community of intestinal bacteria that expresses TMA lyases. One byproduct of the oxidation of TMA that is facilitated by flavin monooxygenases in the liver is TMAO. Researchers have demonstrated that increased levels of TMAO in blood and cerebrospinal fluid contribute to the development and progression of various metabolic, cardiovascular, and cerebrovascular disorders [206]. TMAO level has been positively correlated with neuronal cell aging and the development of neurological disorders [207, 208]. The pathogenesis mediated by TMAO involves the stimulation of inflammatory pathways which include NF-κB, NLRP3 inflammasome, and P38 mitogen-activated protein kinase/Jun N-terminal kinase both in the periphery and brain. By inducing mitochondrial malfunction, oxidative stress, neuronal senescence, and synaptic damage, elevated TMAO levels have been linked to age-related cognitive impairment [206]. Importantly, inhibiting TMAO synthesis with 3,3-dimethyl-1-butanol was demonstrated to alleviate cognitive dysfunction in amyloid precursor protein/presenilin 1 transgenic mice, suggesting a potential relationship between elevated TMAO levels and the etiology of AD [209]. These findings provide evidence for the potential of therapies targeting the removal of harmful compounds produced by gut in the management of neurodegenerative illnesses.

## Impact of GM on CNS Pharmacological Agents

GM can directly affect CNS pharmacology through influencing absorption and metabolism of drugs as well as their therapeutic effects [210] where microbial enzymes and metabolites can influence pharmacokinetics and pharmacodynamics of drugs [211]. Generally, the pharmacokinetic phase can affect the drug’s pharmacological response and the manifestation of its adverse effects. Obviously, GM has the potential to influence drug metabolite generation via synthesis of enzymes that can undergo chemical biotransformation [212]. For example, compared to mice grown in the traditional manner, germ-free mice had significantly lower amounts of CYP3A and higher levels of CYP4A [213]. Clinically, interindividual variations in CYP3A expression significantly affect drug metabolism since the cytochrome CYP3A metabolizes more than half of medicines. Alterations to the composition of microbiota, along with genetic polymorphism, may account for these variations. For instance, nitrazepam, a benzodiazepine, is used to treat anxiety and insomnia. However, it is converted to 7-aminonitrazepam, which is teratogenic when given to pregnant rats. A significant reduction of nitrazepam-induced malformations and a decrease in 7-aminonitrazepam synthesis were observed upon receiving antibiotics prior to nitrazepam administration, suggesting that GM plays a pivotal role in the development of this harmful metabolite [214].

Microbiota can also modulate tacrine hepatotoxicity which is one of the first cholinesterase inhibitors licensed for the management of AD. Experimentally, the increased susceptibility to tacrine-induced transaminitis is attributed to the presence of β-glucuronidase-producing bacteria, such as *Bacteroides* and *Enterobacteriaceae*. These bacteria are responsible for the increased enterohepatic recycling of tacrine-N-glucuronide. Clearly, there has been a significant decrease in the amount of gut bacteria upon administration of vancomycin and imipenem, which impedes the activity of bacterial β-glucuronidase [215]. Drug absorption, the initial step in its pharmacokinetic pathway, can also be influenced by microbiota makeup. Antibiotics like clarithromycin and amoxicillin (used to remove *H. pylori*) affect the absorption of levodopa (L-dopa), the principal drug used for management of PD, causing elevated blood levels of L-dopa compared to non-treated individuals [216]. An in vitro study revealed the binding of L-dopa to *H. pylori*, contributing to the decreased absorption of drug and explaining the increased bioavailability in treated patients [217]. Importantly, more studies are required to clarify this issue as the prolonged use of drugs with antimicrobial effects may have undesirable outcomes, including resistance or harmful adaptations of GM with deleterious consequences to the host [218].

GM metabolites including tryptophan derivatives and glutamate can influence neurotransmission, synaptic plasticity, and neuroinflammation [219, 220]. Metabolites and SCFAs (e.g., propionate and butyrate) generated by GM as well as secondary bile acids are known to regulate cytochrome P450 enzymes, like CYP2C9, CYP1A2, and CYP3A4 through regulation of the epigenetic processes or the nuclear receptor signaling [221, 222]. These processes can affect rate of drug metabolism and thus its therapeutic efficacy and toxicity.

In addition to influencing pharmacokinetic effects of drug, GM can alter its pharmacodynamics [12]. For example, dysbiosis has been correlated to poor response to antidepressants like SSRI in some patients with the presence of reduced taxa involved in tryptophan metabolism and SCFA synthesis which can influence pharmacodynamics of antidepressants [210]. Moreover, GM can affect the pharmacodynamics of drugs through its influence on neurotransmitters and drug targets as well as inflammatory and immunomodulatory pathways [210].

## Impact of CNS Pharmacological Agents on GM

**Fig. 3:**
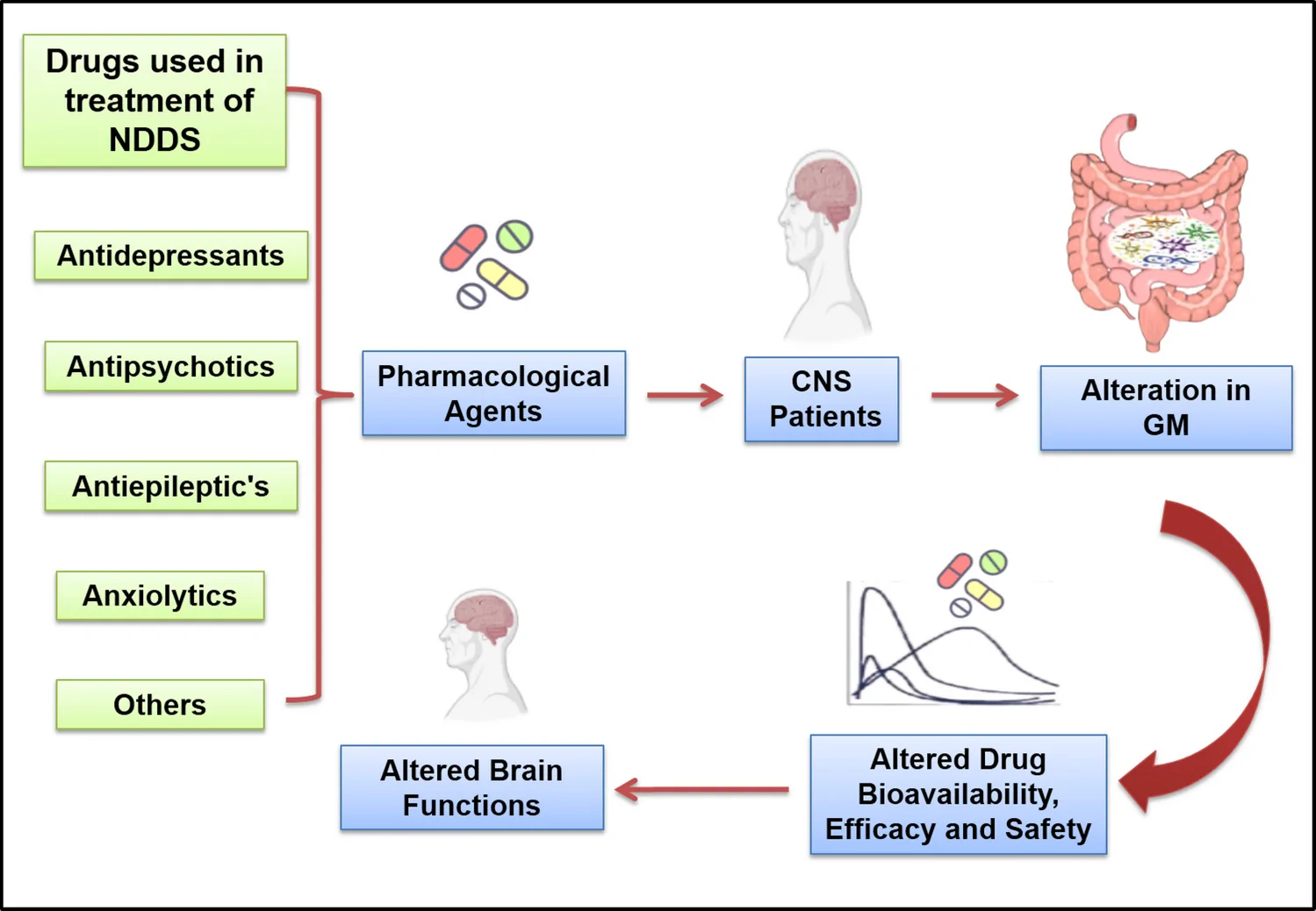
Interactions of the most commonly used CNS drug classes with GM in patients with CNS disorders. CNS, central nervous system; GM, gut microbiota; NDDs, neurodegenerative diseases

GM plays a significant role in modulating safety of drugs through regulating their effectiveness and toxicity [223]. Previous research has shown that microbiota can influence metabolism of drugs, altering their pharmacokinetics and pharmacodynamics or even leading to the creation of deleterious byproducts [224]. Conversely, GM can be influenced by a number of medications, including those used to treat neurodegenerative illnesses, antidepressants, antipsychotics, antiepileptics, anxiolytics, and other medications [225]. Nevertheless, additional research is needed to understand the potential bidirectional interactions between GM and medications and their underlying mechanisms. Some interactions of the most commonly used CNS pharmacological agents with GM in various CNS disorders are summarized in Fig. 3 and Tables 1, 2, 3 and 4.

**Table 1:** Effects of pharmacological agents used in neurodegenerative diseases on GM composition

Model	Drug Types	GM Alterations (↑: Increase, ↓: Decrease)	Reference
Human	Levodopa	*Clostridium*↓	[226]
Human	Levodopa	*Faecalibacterium*, *Roseburia*, *Pseudobutyrivibrio*, *Bacteroides*, *Blautia*, and *Lachnospira*↓ and *Collinsella *↑	[227]
Human	Catechol-O-methyltransferase inhibitors, anticholinergics, carbidopa/levodopa,	*Bifidobacteriaceae,* *Christensenellaceae,* *Lactobacillaceae *and* Verrucomicrobiaceae* ↑	[228]
Mice	Donepezil	*Verrucomicrobia*↑	[229]
Mice	tau antibody 43D	*Restored Gammaproteobacteria* and *Turicibacterales*	[230]

**Table 2:** Effects of antidepressants on GM composition

Model	Drug types	GM alterations (↑ increase, ↓ decrease)	Reference
Mice	Fluoxetine, Escitalopram, Venlafaxine, Duloxetine, Desipramine	β-diversity richness ↓, *Ruminococcus ↓*, and *Adlercreutzia ↓*	[231]
Mice	Fluoxetine	*Lactobacillus* (*Lactobacillus johnsonii*) ↓, *Bacteroidales S24-7* ↓, *Alistipes* ↑, *Lachnospiraceae* ↑, *Lachnoclostridium* ↑, and *Anaerotruncus ↑*	[232]
Human	Escitalopram	Bacterial diversity *↑* (mainly α-diversity)	[233]
Human	SSRIs mainly Paroxetine	*Eubacterium ramulus* ↑	[234]

**Table 3:** Effects of antipsychotics on GM composition

Model	Drug types	GM alterations (↑ increase, ↓ decrease)	Reference
Human	Clozapine, olanzapine, risperidone, quetiapine, asenapine, ziprasidone, lurasidone, aripiprazole, paliperidone, iloperidone	*Lachnospiraceae* ↑ and *Akkermansia* ↓	[235]
Rats	Olanzapine	*Firmicutes* ↑, *Bacteroidetes*↓, *Proteobacteria* ↓, and *Actinobacteria*↓ (more significant in female rats)	[236]
Mice	Olanzapine	*Erysipelotrichi* ↑, *Gammaproteobacteria c*, and *Bacteroidia* ↓	[237]
Rats	Olanzapine	*Bacteroidetes/Firmicutes* ratio ↑ in the gut and altered microbial metabolite SCFAs by *A. muciniphila* ↓ in the gut → levels of 5-HT ↓ → activity of orexotropic neurons ↑ in the hypothalamus via GM-brain axis to induce lipid disturbances	[238]
Rats	Lurasidone	No significant alteration of *Bacteroidetes/Firmicutes* ratio	[239]
Human	Risperidone	*Bacteroidetes/Firmicutes* ratio ↓, *Clostridium* sp. ↑, *Lactobacillus* sp. ↑, *Ralstonia* sp. ↑, and *Erysipelotrichaceae* family ↑	[240]
human	Risperidone	*Bifidobacterium* spp. ↑, *Escherichia coli* ↑, *Clostridium coccoides* ↓, and *Lactobacillus* spp. ↓	[241]
Mice	Risperidone	*Erysipelotrichaceae:(Allobaculum, Turicibacter* spp.*)* ↑, *Mollicutes class: (Anaeroplasma* spp.*)* ↑, *Bacteroides* ↑, *Alistipes* spp. ↓, *Akkermansia* spp. ↓, and *Lactobacillus* spp. ↓	[242]
Rats	Aripiprazole	*Bacterial* diversity ↑, *Firmicutes* ↑, *Peptostreptococcaceae* ↑, *Clostridiaceae* ↑, *Ruminococcaceae* ↑, *Ruminococcus 1*↓	[243]
Rats	Lithium	Bacterial diversity ↑, *Actinobacteria* ↑, *Bacteroides* ↓, *Peptostreptococcaceae* ↑, *Clostridiaceae* ↑, *Ruminococcaceae* ↑, and *Ruminococcus 1*↓	[243]

**Table 4:** Effects of antiepileptics and other drugs on GM composition

Model	Drug types	GM alterations (↑ increase, ↓ decrease)	Reference
Rats	Valproate	Bacterial diversity ↑, *Actinobacteria* ↑, *Bacteroides* ↓, *Peptostreptococcaceae* ↑, *Clostridiaceae* ↑, *Ruminococcaceae* ↑, and *S24-7 uncultbact* ↓	[243]
Mice	Topiramate	*Lactobacillus johnsonii*	[244]
Rats	Ketamine	*Lactobacillus*, *Turicibacter*, and *Sarcina ↑* *Mucispirillum* and *Ruminococcus ↓*	[245]

### Drugs Used in Treatment of Neurodegenerative Diseases and GM

Currently, drug therapy remains the main approach for managing neurodegenerative diseases. Actions and functions correlated with GM byproducts or metabolites can be influenced by medications, either directly or indirectly [246]. One example is the impact on GM composition and microbial diversity caused by AD medications like acetylcholinesterase inhibitors and N-methyl-D-aspartate receptor antagonists. It has been suggested that memantine enhanced the capture and bactericidal activity of neutrophils produced by pathogenic *Escherichia coli* in the gut [247, 248]. AD drugs can induce alterations in composition of GM and thus affecting their derived metabolites, modulating the stimulation of vagus nerve, ameliorating neuroinflammation and neuronal plasticity via the brain-gut axis, and leading to beneficial effects on cognitive functions. Microbiome of the saliva has been found to be more affected by acetylcholinesterase inhibitors than microbiota of the gut, which is an interesting finding [249]. In a mouse model of AD, an increase in *Verrucomicrobia* and a change in the relative abundance of 12 taxa, including *Boltia* and *Akkermansia*, were seen in the donepezil-treated group compared to the control group [229].

Immunotherapy has recently been employed in AD to target the primary pathophysiological processes, specifically Aβ and tau protein-related plaques [250]. One mechanism of action of these medicines is to modify the GM. When compared to untreated mice, transgenic mice with AD showed a typical restoration of the dysregulated patterns of *Gammaproteobacteria* and *Turicibacterales* after immunization with 43D tau antibody [230]. In PD, a study assessing 46 patients after being treated with L-dopa revealed reduction of abundance of genera *Roseburia*, *Faecalibacterium*, and *Pseudobutyrivibrio*, and species relating to *Blautia*, *Bacteroides*, and *Lachnospira*, together with elevation of *Collinsella*, when compared to untreated patients [227]. Using catechol-O-methyltransferase inhibitors, particularly entacapone, also increased *Actinobacteria*, *Lactobacillaceae*, *Porphyromonadaceae*, and *Proteobacteria* abundance in addition to reducing *Firmicutes* abundance [251, 252]. Specifically, one of the most common SCFAs produced by GM, butyrate, was found to be negatively correlated with entacapone use [253]. Recently, entacapone was reported to interfere with the balance of gut flora via sequestration of iron [254].

Furthermore, a reduction in the abundance of *Rombolia* spp. has been observed in an experimental evaluation of the effects of dopamine agonists (such as pramipexole and ropinirole) in conjunction with levodopa-carbidopa. In the pramipexole-treated group, a reduction in abundance of *Romboutsia* spp. was observed in the proximal small intestine and *Lachnospiraceae* spp. in the ileum, while in both pramipexole- and ropinirole-treated groups, an increase in abundance of *Lactobacillus* spp. was revealed [255]. Interestingly, a negative correlation exists between *Lactobacillus* abundance and plasma L-dopa levels [255], where the abovementioned changes in *Lactobacillus* or *Lachnospiraceae* were observed in healthy rats treated with dopamine agonists and L-dopa [45]. Moreover, decreased levels of SCFA-producing bacteria (e.g., *Lachospiraceae*) and elevated levels of *Lactobacillus* or *Bifidobacterium* were positively correlated with increasing doses of L-dopa, confirming the idea that these changes in GM may be the result of PD medication rather than the disease itself [256]. Importantly, elevated levels of some families of bacteria such as *Bifidobacteriaceae*, *Christensenellaceae*, *Lachnospiraceae*, *Lactobacillaceae*, *Pasteurellaceae*, and *Verrucomicrobiaceae* have been demonstrated upon the use of various PD medications, including catechol-O-methyltransferase inhibitors, anticholinergics, and carbidopa/L-dopa [228]. The effects of pharmacological agents used in neurodegenerative diseases on GM are summarized in Table 1.

### Antidepressants and GM

As recently shown in psychiatric studies, the gut-brain axis connects the emotional and cognitive centers of brain to the intestinal tract function [257, 258]. Antidepressants, such as SSRIs, tricyclic antidepressants, and monoamine oxidase inhibitors, have shown impacts on gut function and microbiota composition, which can affect mood and mental health [259].

More than amitriptyline, fluoxetine considerably could change the total GM profile in rats subjected to prolonged unpredictable mild stress, with increased *Bacteroidetes* and decreased *Firmicutes* relative abundance [260]. Importantly, fluoxetine decreased the abundance of pathogens like *Escherichia coli* and *Shigella* as well as conditional pathogens like *E. coli*, *Vagococcus*, and *Aerococcus*, suggesting that CNS acting drug could alter the GM [261]. In addition to alleviating depression, fluoxetine raises gut flora abundance of *Lactobacillus*, which in turn affects ENS-mediated serotonin 1 A and 2 C as well as melanocortin-4 receptor expressions [262]. Furthermore, a study conducted in Netherlands found that individuals with irritable bowel syndrome who used paroxetine had a markedly higher abundance of *Eubacteriumramulus* compared to control subjects [234]. Additionally, venlafaxine reduced depression symptoms and controlled GM composition in cuprizone-treated mice through increasing the abundance of *Lactobacillus* and *Bifidobacterium* [263].

Clearly, the interaction between GM and ketamine was discovered to mediate some of its antidepressant effects, leading to increase of 3.3-, 26-, and 42-fold in *Lactobacillus*, *Turicibacter*, and *Sarcina*, respectively and decrease of approximately 2.6- and 26-fold in *Mucspirillum* and *Rhinococcus*, respectively [245]. Moreover, animal models of depression have shown that administration of some species of *Lactobacillus* alleviated depressive-like behavior, suggesting that low levels of *Lactobacillus* and *Turicibacter* are connected with a variety of CNS disorders [264, 265]. On the other hand, a change in microbial communities towards dysbiosis, with decreased relative abundance of *Lactobacillus johnsonii* and *Bacteroidales S24-7*, may be responsible for some of the known side effects of antidepressants, like weight loss upon fluoxetine supplementation [232]. The effects of antidepressants on GM are summarized in Table 2.

### Antipsychotics and GM

Metabolic disorders that are related to changes in GM composition or gut dysbiosis are frequently linked to the use of atypical antipsychotics. A decrease in *A. muciniphila* level was reported by Flowers and colleagues in the gut upon the use of atypical antipsychotics [235]. Specifically, olanzapine decreased the level of *A. muciniphila* and led to changes in the amounts of SCFAs due to increase in the *Firmicutes*/*Bacteroidetes* ratio in the gut. These changes cause 5-HT levels to drop, which in turn stimulates the activation of exotropic neurons in the hypothalamus through the GM-brain axis, causing abnormalities in lipid metabolism. Increased pro-inflammatory status and alterations in blood glycaemic and lipid levels are related to microbiome alterations together with metabolic dysfunction [238]. Conversely, lurasidone, an atypical antipsychotic, shows no discernible effects on weight gain or metabolism; rather, it modulates the GM favorably by increasing its alpha diversity and maintaining baseline ratios of *Firmicutes* to *Bacteroidetes* [239].

In a similar vein, patients with increased body mass index were more likely to have a decrease in the relative abundance of *Bacteroidetes* and an increase in the relative abundance of the *Firmicutes* phylum after prolonged use of risperidone, a commonly used second generation antipsychotic [240]. Yet, another study corroborated this finding, showing that 24 weeks of risperidone medication led to a substantial rise in body mass index as well as changes in fasting blood-glucose, triglyceride, and low density lipoprotein levels with a corresponding elevation in the amount of fecal *Bifidobacterium* spp. an *E. coli* as well as marked decline in the abundance of *Clostridium difficile* and *Lactobacillus* species in feces [241].

Experimentally, aripiprazole, another atypical antipsychotic, significantly altered the microbiota composition after 4 weeks of treatment, including considerable reductions in relative abundance of several taxa, such as *Ruminiclostridium*, *Clostridium*, *Intestinibacter*, and *Eubacterium coprostanoligens*. The primary saturated fatty acids (butyrate, acetate, and propionate) were reported to be metabolically reduced as result of these microbial alterations [243]. Lithium, a mood stabilizer, was also shown to alter GM composition by elevating *Ruminococcaceae* relative abundance and decreasing *Bacteroides* level [243]. The effects of antipsychotics on GM are summarized in Table 3.

### Antiepileptics and GM

Growth of gut bacterial species and consequently the host response are both affected by the use of anti-seizure medications [266]. There is accumulating evidence that refractory epileptic patients have an imbalanced GM, with drug-sensitive epilepsy patients showing a marked increase in *Saccharimonadales* and *Peptoclostridium*, while anti-epileptic non-responders are being recognized with increased *Vibrionaceae* and *Rhodobacteraceae*. As a predictive biomarker for drug resistance in epilepsy, gut dysbiosis may contribute to resistance to anti-epileptics by increasing the metabolic pathways involved in vanillin and taurine degradation in comparison to controls [267]. Some anti-epileptic drug excipients, such as propyl paraben and other artificial sweeteners, have also been correlated with gut dysbiosis in patients using carbamazepine, lamotrigine, or topiramate [266].

Further evidence shows that valproate could induce changes in GM which might contribute to the metabolic dysfunction, weight gain, and changes in biochemical indices [268]. Interestingly, 6-(4-chlorophenoxy)-tetrazolo [5,1-a] phthalazine (Q808) was revealed to successfully alleviate seizures in a chronic pentylenetetrazole-kindled model of rats. This was achieved by resolving gut dysbiosis, which led to increase in bacteria-producing SCFAs. Additionally, Q808 improved acetylcholine levels in the hippocampus while decreasing 3-methoxytyramine, glutamine, and 5-hydroxyindole acetic acid [269]. The effects of antiepileptics and other drugs on GM are summarized in Table 4.

## Combined Regimens of Microbiota-Based Treatments with Classic CNS Pharmacological Agents in Various CNS Disorders

Through changes in gut-brain axis, GM modulation has revealed to enhance the efficacy of traditional pharmacological agents treating various CNS disorders [270, 271]. For example, the co-administration of *Bifidobacterium animalis* subsp. *Lactis* Probio-M8 enhanced clinically the efficacy of benserazide and dopamine agonists in PD patients through improvement of patient sleep quality and alleviation of gastrointestinal symptoms and anxiety [270]. Probiotic supplementation with fluoxetine also considerably reduced depression severity in patients with MDD, according to a prior study where this probiotic included *Lactobacillus helveticus* R0052 and *Bifidobacterium longum* R0175 [271]. Moreover, according to Nikolova et al. (2023), using probiotics containing 14 strains in conjunction with antidepressants significantly reduced depression and anxiety symptoms [272]. Probiotic through microbiota modification may impact mood via the gut-brain axis, as shown in a study by Schaub et al. (2022) which revealed that increased *Lactobacillus* levels were linked to lower depression symptoms [275]. In addition, fluoxetine, when used together with synbiotics containing probiotics (*Lactobacillus casaei*, *Lactobacillus Acidofilus*, *Lactobacillus Bulgarigus*, *Lactobacillus rhamnosus*, *Bifidobacterium Breve*, *Bifidobacterium Longum*, and *StreptococusThermophilus*) and prebiotics (Fructooligosaccharide), succeeded to significantly reduce the severity of depression when compared to use of fluoxetine alone [273].

Arifdjanova et al. (2021) found comparable results with Bac-Set Forte, a 14-strain probiotic which significantly increased efficacy of escitalopram with improvements of depressive symptoms and neuroimmune endocrine markers. These results support the idea that probiotics have an effect on the gut-brain axis and the inflammatory pathways in depressive disorders [274]. Additionally, the use of conventional antidepressant in combination with probiotic cocktail containing strains from the genera *Streptococcus*, *Bifidobacterium*, and *Lactobacillus* fastened symptoms recovery and maintained the abundance of the *Prevotella* enterotype, which is markedly decreased in patients using conventional therapy alone [275]. Furthermore, when compared to the standard treatment, the intensity of depression was significantly reduced when *Clostridium butyricum* MIYAIRI 588 was used in combination with conventional antidepressants such as flvoxamine, paroxetine, escitalopram, duroxetine, and sertraline [276]. Minocycline, when used in conjunction with antidepressants, also improved patients’ cognitive abilities, social lives, and ability to work [277]. Interestingly, topiramate and *Lactobacillus johnsonii* reduced the incidence of pentylenetetrazole-related seizures more effectively than either medicine alone. Increased butyrate and butyrate-producing Lachnospiraceae levels, which in turn could elevate the GABA/glutamate ratio in the cortex, might explain these outcomes [277].

Researchers have shown that combining olanzapine with a freeze-dried probiotic sachet containing *Streptococcus thermophiles* in addition to various *Bifidobacterium* and *Lactobacillus* sp. could alleviate metabolic syndrome symptoms caused by olanzapine through changing GM composition. A reduction of *Firmicute* abundance and elevation of *Bacteroidetes*, *Actinobacteria*, *Prevotella*, *Blautia*, *Bacteroides*, *Bacteroidales*, and *Ruminococcaceae* abundance were among these changes, which also involved the modulation of neurotransmitter levels (serotonin and dopamine levels) and the maintenance of integrity of the gut barrier [244]. Importantly, probiotics concurrently used with olanzapine effectively reduced insulin resistance with no noticeable impact on weight gain in first-episode schizophrenia patients who have never taken drugs before [278]. Similarly, probiotics could alleviate the associated dysbiosis occurring with the use of psychotropics, as shown when *Lactobacillus rhamnosus* and *Bifidobacterium longum* were added to aripiprazole. The addition of probiotics to this combined regimen revealed protective effects through attenuating psychotropics-related alteration of microbiota composition and thus increasing the concentrations of SCFAs [279].

**Fig. 4:**
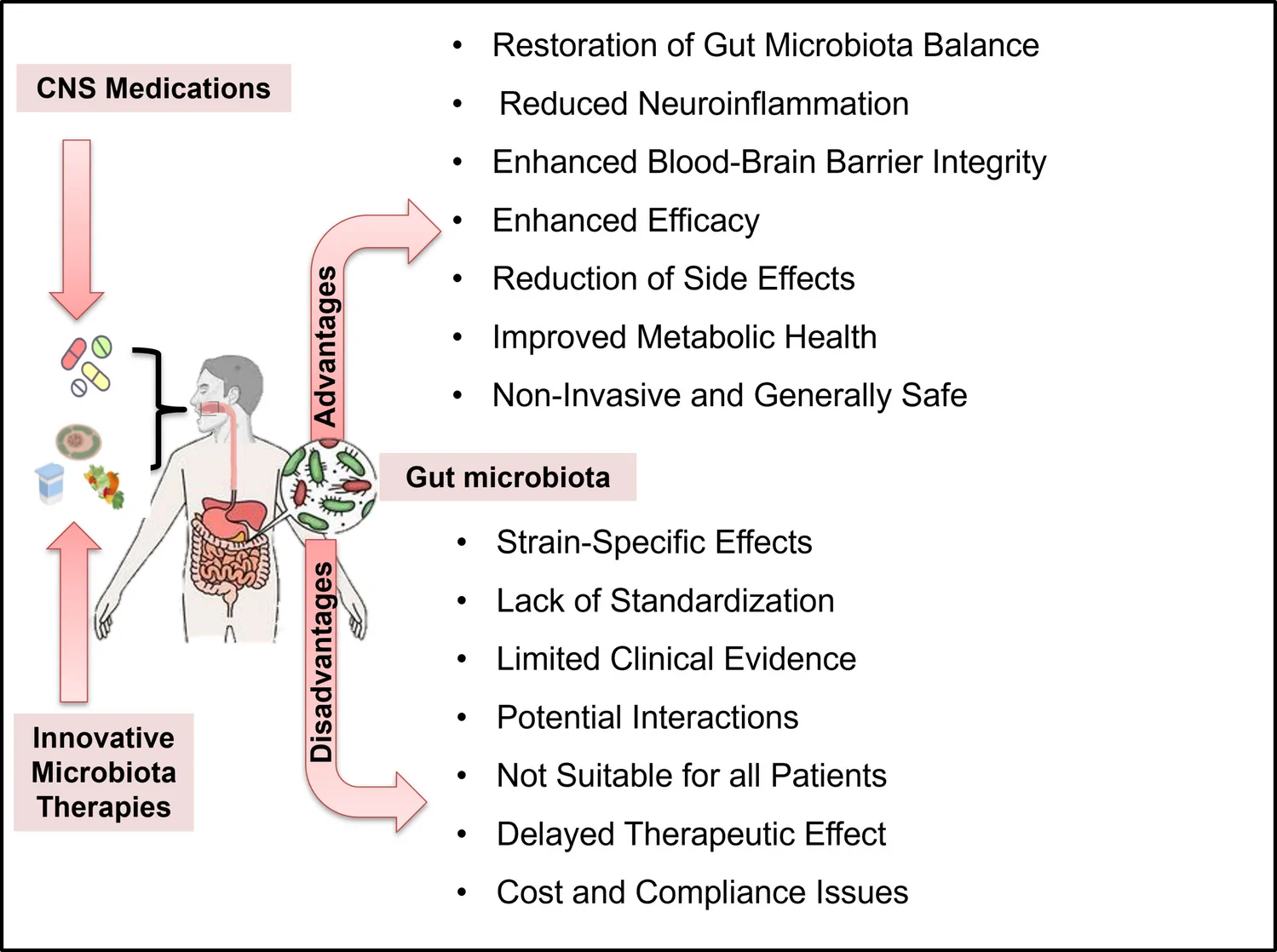
Advantages and disadvantages of combining innovative microbiota therapies with conventional CNS drugs in the management of CNS disorders

In brief, the benefits and drawbacks of these novel combined regimens in various CNS disorders are illustrated in Fig. 4, which suggest that probiotic co-treatment may increase the effectiveness or decrease the adverse effects of traditional therapy through resolving gut dysbiosis and its related negative impacts on both metabolism and function of neurotransmitters.

Despite the promising outcomes of probiotics use as an adjuvant therapy in the management of various CNS disorders, several drawbacks are present including small-scale trials and heterogeneous microbiota-based therapies with inconsistent results among different populations. These factors are considered a major barrier to clinical translation in addition to absence of standardized formulations of microbiota-based therapies, approved regimen and dosing scale, and data for safety upon prolonged use [280, 281]. Thus, to allow reproducibility of results and promote regulatory approval, future studies should concentrate on large well-designed and regulated trials with standardized microbiota-based preparations and with integration of multi-omics approaches and utilization of biomarkers to detect the exact response [281]. Emerging evidence suggests that personalized biomarkers could help predict who can benefit most from probiotic interventions. For example, the presence of balanced microbiota diversity, elevated levels of *Bifidobacterium* and *Lactobacillus*, and reduced level of pro-inflammatory taxa are correlated with superior outcomes in psychiatric and neurological disorders [282, 283]. Similarly, the detection of promising metabolomic profiles, like increased SCFAs and controlled tryptophan metabolism, is linked with improved mood and cognitive function [284, 285]. Integration of multi-omics approaches into well-designed clinical trials will be important for identification of biomarkers that indicate the patient’s response to specific personalized microbiota-based therapy [286].

## Future Perspectives and Challenges

GM can be a therapeutic target in treatment and prevention of different CNS disorders [287]. Unhealthy diet can worsen gut dysbiosis and lead to the production of harmful byproducts, which in turn can interrupt vital metabolic processes and start a cascade of diseases, including disorders affecting the CNS [288]. Conversely, GM is positively modulated by healthy diets like Mediterranean diets and other dietary interventions, leading to increase in its diversity with associated neuroprotective benefits [289]. Prebiotics, probiotics, or FMT derived from healthy volunteers may also modulate microbial composition, re-establish host homeostasis, and positively impact CNS function. It is worth mentioning that the neuroprotective effects of combining prebiotics and probiotics are superior to those of using either of them alone [290].

Due to the fact that GM varies greatly from patient to patient, it is very difficult to assess the effects of microbial composition and function in clinical settings. Furthermore, research is needed to evaluate a step-by-step microbiome change during the development and progression of different CNS disorders and in response to use of conventional medications [222, 291]. When evaluating the variations of pharmacological responses in patients, it is important to consider GM composition. Phytochemicals and various dietary supplements can be utilized by GM to influence their diversity and the generated metabolic byproducts and hence affecting the severity of diseases and their progression as well as the outcome of the utilized medications and their related side effects [292]. Moreover, the therapeutic efficacy of the used drugs can be affected by altering or reshaping GM because it is engaged in the intestinal prehepatic metabolism of these medications [293, 294]. Analysis of the composition of GM before prescribing the appropriate medications can also positively affect the management of these diseases. Nevertheless, there are still obstacles to overcome when trying to use the experimental results in clinical settings, mainly because GM is so complicated and due to variations between laboratory models and real clinical cases [295]. Importantly, it may not be practical to use antibiotics as a tool to evaluate the function and impact of specific GM for an extended period of time in clinical settings because of risks of antibiotic resistance and the need of maintaining a balanced GM for host health [296, 297]. On the other hand, modifying GM with probiotics, prebiotics, or FMT from healthy volunteers can be effective ways to increase therapeutic efficacy of the current utilized medications and decrease their side effects [290]. Clearly, both metabolomics and GM profiling may reveal the mechanisms or pathways by which GM interacts with the host, leading to development of more effective treatments and precise dosages for disease management [298].

Progress in microbiome profiling can also permit the evolution of individualized or personalized microbiota-based interventions according to each patient’s unique GM composition. Importantly, personalized microbiota-based approaches could increase efficacy of utilized treatments, decrease variability in results, and reduce adverse effects [299]. However, it is still applicable on a small-scale and on limited clinical phenotypes like bowel disorders [300] due to high cost, long timescales required for accurate microbiome analyses, and presence of insufficient standardized laboratory techniques [300, 301]. Notably, the utilization of personalized biomarkers from microbiome profiling could predict the possible benefits from use of specified microbiota-based interventions.

## Conclusion

Alterations of microbiota composition play significant role in the development and progression of different neurological disorders. However, there is still a lack of knowledge regarding the relationships between host microbiota and influence on CNS disorder progression and drug response. Patient’s microbiome can affect pharmacokinetics or pharmacodynamics of drugs influencing the patients’ response to traditional therapies. On the other hand, the diversity or composition of GM can be affected by these medications. All these aspects can impact the management of different CNS disorders and the risk of their progression. Importantly, GM modulation with dietary interventions, prebiotics, probiotics, and FMT can amplify the efficacy of the used medications or eliminate their side effects in addition to their positive impacts on the disease status and its associated complications. However, more studies are still required to evaluate extensively the mechanisms by which GM modulates CNS risk and affects response to medications before the clinical translation of this approach. Collectively, modulation of GM can offer a new therapeutic target to boost outcomes of the existing CNS medications, ultimately leading to better health outcomes in patients.

## Data Availability

No datasets were generated or analysed during the current study.

## Abbreviations

Aβ: Amyloid beta
ALS: Amyotrophic lateral sclerosis
BDNF: Brain-derived neurotrophic factor
CNS: Central nervous system
DHA: Docosahexaenoicacid
EPA: Eicosapentaenoic acid
ENS: Enteric nervous system
FMT: Fecal microbiota transplantation
GABA: Gamma-aminobutyric acid
GM: Gut microbiota
IL: Interleukin
L-dopa: Levodopa
LPS: Lipopolysaccharide
MDD: Major depressive disorder
MOS: Mannooligosaccharides
NLRP3: NOD-like receptor protein 3
NF-κB: Nuclear factor kappa B
PD: Parkinson’s disease
PM: Polymannuronic acid
SSRIs: Selective serotonin reuptake inhibitors
SCFA: Short-chain fatty acid
TLR4: Toll-like receptor 4
TMA: Trimethylamine
TMAO: Trimethylamine N-oxide
TNF-α: Tumor necrosis factor alpha
α-Syn: α-Synuclein
